# Going digital to boost safe and sustainable materials innovation markets. The digital safe-and-sustainability-by-design innovation approach of the PINK project

**DOI:** 10.1016/j.csbj.2025.03.019

**Published:** 2025-03-15

**Authors:** Thomas E. Exner, Joh Dokler, Steffi Friedrichs, Christian Seitz, Francesca L. Bleken, Jesper Friis, Thomas F. Hagelien, Francesco Mercuri, Anna L. Costa, Irini Furxhi, Haralambos Sarimveis, Antreas Afantitis, Antonino Marvuglia, Gustavo M. Larrea-Gallegos, Tommaso Serchi, Angela Serra, Dario Greco, Penny Nymark, Martin Himly, Karin Wiench, Nico Watzek, Eva-Kathrin Schillinger, Jérôme Gavillet, Iseult Lynch, Andreas Karwath, Alexe L. Haywood, Georgios V. Gkoutos, Roland Hischier

**Affiliations:** a Seven Past Nine d.o.o., Hribljane, Cerknica 1380, Slovenia; b Seven Past Nine GmbH., Rebacker 6, Schopfheim 79650, Germany; c AcumenIST SRL, Rue Fétis 19, Bruxelles 1040, Belgium; d SINTEF AS, Strindvegen 4, Trondheim 7034, Norway; e SINTEF Ocean AS, Paul Fjermestads Veg 59, Trondheim 7052, Norway; f Istituto per lo Studio dei Materiali Nanostrutturati (ISMN), Consiglio Nazionale delle Ricerche, Via P. Gobetti 101, Bologna 40128, Italy; g Istituto di Scienza, Tecnologia e Sostenibilità per lo Sviluppo dei Materiali Ceramici. Consiglio Nationale Delle Ricerche (CNR-ISSMC), Via Granarolo 64, Faenza 48018, Italy; h School of Chemical Engineering, National Technical University of Athens, 9 Heroon Polytechniou, Athens 15780, Greece; i Novamechanics Limited, Digeni Akrita 51, Nicosia 1070, Cyprus; j Luxembourg Institute of Science and Technology, 5, avenue des Hauts-Fourneaux, Esch-sur-Alzette 4362, Luxembourg; k Finnish Hub for Development and Validation of Integrated Approaches (FHAIVE), Faculty of Medicine and Health Technology, Tampere University, Tampere 33520, Finland; l Division of Pharmaceutical Biosciences, Faculty of Pharmacy, University of Helsinki, Helsinki 00790, Finland; m Institute for Environmental Medicine, Karolinska Institutet, Nobels Väg 5, Stockholm 17177, Sweden; n Department of Biosciences & Medical Biology, Paris Lodron Universität Salzburg, Hellbrunnerstrasse 34, Salzburg 5020, Austria; o BASF SE, Carl Bosch Str. 38, Ludwigshafen am Rhein 67056, Germany; p Innovative Advanced Materials Initiative, Rue de Ransbeek 310, Bruxelles 1120, Belgium; q School of Geography, Earth and Environmental Sciences, University of Birmingham, Edgbaston, Birmingham B15 2TT, United Kingdom; r Centre for Environmental Research and Justice, University of Birmingham, Edgbaston, Birmingham B15 2TT, United Kingdom; s Department of Cancer and Genomic Sciences, University of Birmingham, Edgbaston, Birmingham B15 2TT, United Kingdom; t Centre for Health Data Science, University of Birmingham, Edgbaston, Birmingham B15 2TT, United Kingdom; u Advancing Life Cycle Assessment Group, Swiss Federal Laboratories for Materials Science and Technology, Lerchenfeldstrasse 5, St. Gallen 9014, Switzerland

**Keywords:** Advanced materials, Safe-and-sustainable-by-design, Computational modelling and simulations, Functionality modelling, Safety assessment, Life cycle assessment, Life cycle costing

## Abstract

In this innovation report, we present the vision of the PINK project to foster Safe-and-Sustainable-by-Design (SSbD) advanced materials and chemicals (AdMas&Chems) development by integrating state-of-the-art computational modelling, simulation tools and data resources. PINK proposes a novel approach for the use of the SSbD Framework, whose innovative approach is based on the application of a multi-objective optimisation procedure for the criteria of functionality, safety, sustainability and cost efficiency. At the core is the PINK open innovation platform, a distributed system that integrates all relevant modelling resources enriched with advanced data visualisation and an AI-driven decision support system. Data and modelling tools from the, in large parts, independently developed areas of functional design, safety assessment, life cycle assessment & costing are brought together based on a newly created Interoperability Framework. The PINK *In Silico* Hub, as the user Interface to the platform, finally guides the user through the complete AdMas&Chems development process from idea creation to market introduction. Guided by two Developmental Case Studies, the process of building of the PINK Platform is iterative, ensuring industry readiness to implement and apply it. Additionally, the Industrial Demonstrator programme will be introduced as part of the final project phase, which allows industry partners and especially small and medium enterprises (SMEs) to become part of the PINK consortium. Feedback from the Demonstrators as well as other stakeholder-engagement activities and collaborations will shape the platform’s final look and feel and, even more important, activities to assure long-term technical sustainability.

## Graphical Abstract

**Figure:**
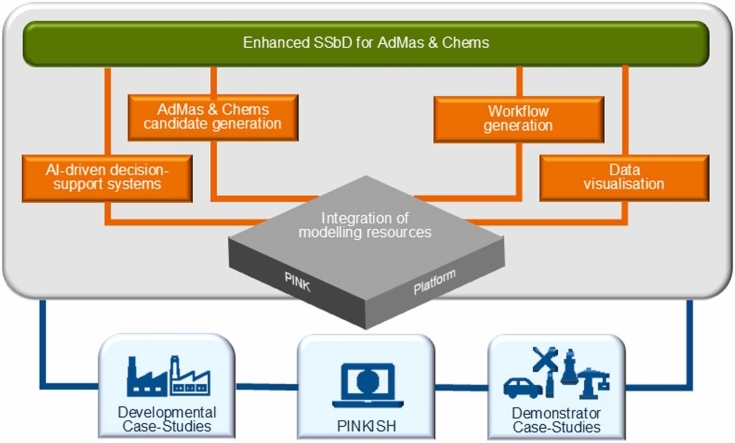

## 1 Introduction

The European Union’s Green Deal [1] and the proposed Clean Industrial Deal [2] strive to make Europe the first climate-neutral continent by setting a path for a transformation of its economy into a sustainable, climate-neutral and circular, competitive and resilient powerhouse that is just and socially fair. This ambitious vision is underpinned by pivotal EU frameworks, e.g. the EU Chemical Strategy for Sustainability (CSS) [3], Communication on Advanced Materials for Industrial Leadership [4], the Zero Pollution Action Plan [5], the Circular Economy Action Plan [6], the European Climate Law [7] and other specific policies. Together, these policies are driving both environmental ("green") and technological ("digital") innovations, labelled as the twin green and digital transformation [8]. To achieve this transformation, many innovation-driven markets including (but not limited to) healthcare, construction, new energies, transportation, electronics and agriculture, urgently require innovative materials as outlined in the Advanced Materials Initiative 2030 (AMI2030) roadmap [9], which will be further implemented in the Innovative Advanced Materials for Europe (IAM4EU) Partnership [10]. This central public-private partnership (PPP) for Advanced Materials in Europe will be operationalised by the PINK consortium partner Innovative Advanced Materials Initiative (IAM-I).

Advanced materials and chemicals (AdMas&Chems) must provide the expected new or improved functionality and, at the same time, be both safe and sustainable, i.e. they need to be developed in line with Safe-and-Sustainable-by-Design (SSbD) concepts.

When developed properly, AdMas&Chems will play a crucial role in:

• Reducing dependence on critical raw materials;
• Lowering energy consumption and greenhouse gas emissions;
• Achieving a toxic-free environment.

The EU Joint Research Centre (JRC) was tasked to develop a framework for guiding industry on how to improve safety and sustainability performance along the complete value chain and therefore also the AdMas&Chems life cycle [11], [12], [13], [14]. The resulting reports and guidance notes led to the adoption of the SSbD approach in the Commission Recommendation of 8 Dec. 2022 [15], hereafter referred to as the “SSbD Framework”.

Nanosafety research driven in part by partners from the PINK consortium developed and tested Safe-by-Design (SbD) principles [16], [17], [18], [19], [20], [21], [22], [23], [24], [25], [26] through a variety of European projects, such as e.g. NanoReg [27], ProSafe [28] and NanoReg2 [19], [29]. The latter made first steps towards a combined safety and sustainability assessment [30], which were then further refined for nanomaterials in projects like ASINA [31], [32], SUNSHINE [33], [34], DIAGONAL [35], HARMLESS [36] and MACRAMÉ [37], [38]. As already been recommended in the SbD approach, the SSbD Framework follows a hierarchical approach consisting of two high-level components (the 1st level of the hierarchy), which are executed in an iterative manner: (i) the (re-)design phase, and (ii) the safety and sustainability assessment phase. For the (re-)design, the SSbD Framework mainly provides a list of SSbD design principles and then refers to the stage-gate process [39] as an established approach into which industrial innovation is structured.

**Fig. 1:**
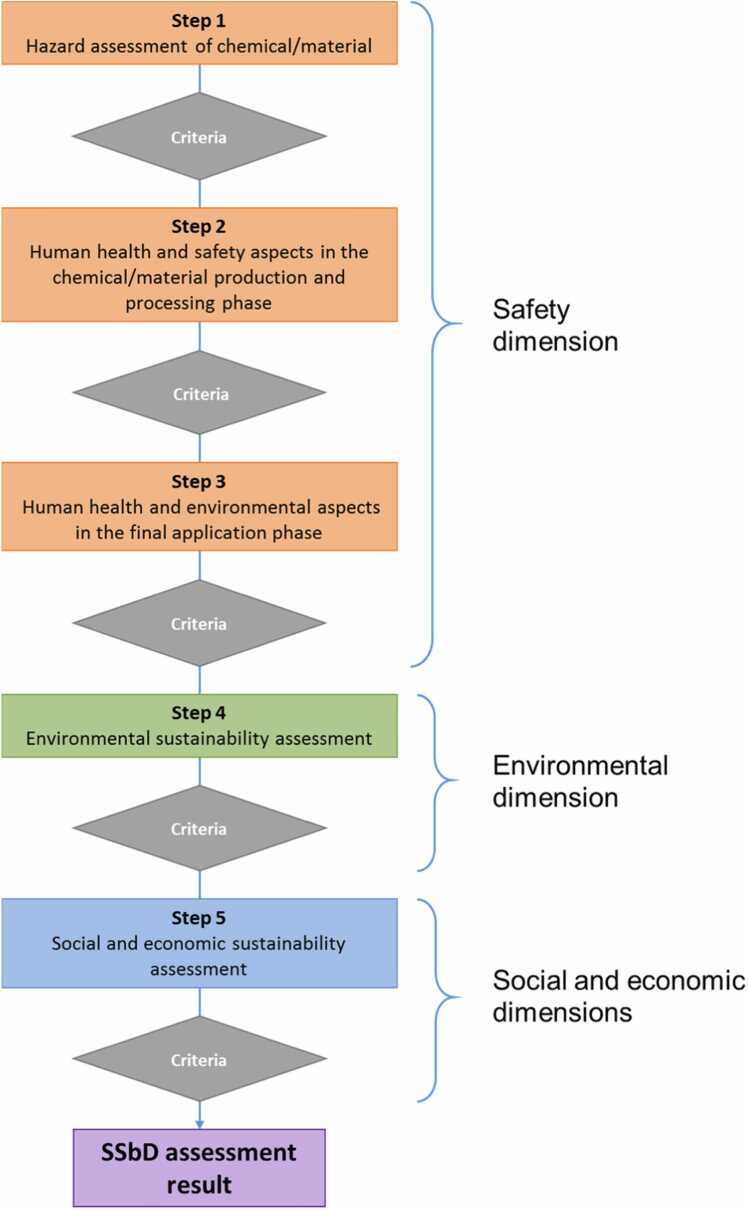
The 5 steps or dimensions of the stage-gate approach as proposed by the SSbD Framework, reproduced from [12].

Within SSbD, the assessment phase is then based on a defined set of criteria (the 2nd level of hierarchy) to evaluate the extent of compliance with the SSbD Framework [11], [12]. This assessment is further broken down into five key steps or dimensions (see Fig. 1);

• Step 1: Hazard assessment of chemical/material;
• Step 2: Human health and safety aspects in the chemical/material production and processing phase;
• Step 3: Human health and environmental aspects in the application phase;
• Step 4: Environmental sustainability assessment;
• Step 5: Socio-economic sustainability assessment.

Although the word “step” implies a consecutive execution, the SSbD Framework explicitly allows for the possibility to run them in parallel. The final (3rd) hierarchy level encompasses the specific assessment criteria, also called aspects or indicators, which are tailored to each of the 5 steps.

There is consensus that covering the demand of AdMas&Chems across all material innovation markets and especially the implementation of safety and sustainability at the earliest possible stage of AdMas&Chems development is only possible through the extensive use of computational modelling and simulation approaches [13], [14]. These approaches play a critical role in addressing data gaps across all SSbD dimensions. Furthermore, AI-driven decision support systems (DSS) are seen as essential for making informed decisions leveraging all available data [40], [41]. This article described how PINK [42] directly addresses the urgent need for such digital solutions to foster further implementation of SSbD-conform materials development in industrial settings by providing advanced and interoperable tools (computational modelling, simulation, and AI-driven decision support) applicable already at early stages of material innovation and accessible.

## 2 Project description

The PINK project (Provision of Integrated Computational Approaches for Addressing New Markets Goals for the Introduction of Safe-and-Sustainable-by-Design Chemicals and Materials, Grant Agreement No. 101137809) was funded under the funding call HORIZON-CL4–2023-RESILIENCE-01–23: Computational models for the development of safe and sustainable by design chemicals and materials and received additional support from the Swiss State Secretariat for Education, Research and Innovation (SERI) and the United Kingdom Research and Innovation (UKRI) via Innovate UK. Its consortium consists of 6 research institutes from Italy, Luxembourg, Norway (2x), Sweden and Switzerland, 4 universities from Austria, Finland, Greece, and the UK, 4 small and medium enterprises (SMEs) from Belgium, Cyprus, Germany, and Slovenia (coordinator), one innovative large industry player and one industry association. Together they form a highly interdisciplinary team of leading experts across all SSbD dimensions and innovation stages, i.e. materials development and design, materials modelling, chemical/material safety assessment, life cycle assessment, circularity, as well as business development and market research, and relevant digital technologies ranging from data-driven and physics-based modelling, knowledge representation, artificial intelligence, data management and data science, semantic interoperability, and open innovation platform and infrastructure design and implementation. These profiles were selected to address the challenges fully computational workflow developments with focus on industrial requirements pose. In consequence, the PINK methodological approach pursues the following eight objectives:

1. Conceptualise and REFINE PINK’s research and innovation strategy for providing digital solutions based on industry requirements and stakeholder feedback;
2. DEVELOP innovative modelling and simulation approaches addressing industrial SSbD needs;
3. INTEGRATE these tools to make them accessible to SMEs and industry through the PINK open innovation platform and its user-facing PINK *In Silico* Hub (*PINKISH*);
4. VALIDATE the platform by applying it to PINK Developmental Case Studies and Industrial Demonstrators provided by SMEs and large industries;
5. Address REAL-LIFE industry needs:
  a. Assessment challenges in biodegradability of co-polymers in aqueous dispersions;
  b. Long-term aquatic environmental effects of organic UV-blockers in cosmetics and personal care products;
6. IMPLEMENT open science and FAIR (Findable, Accessible, Interoperable, Reusable) [43], [44], [45], [46] principles contributing to establishing a European AdMas&Chems data, modelling and software ecosystem;
7. STRENGTHEN knowledge transfer through collaboration and exploiting synergies across academia, industry, and research institutions;
8. BOOST the innovative capacity of SMEs and industry, enhancing their agility to respond to external and internal influences.

By addressing the objectives described above, PINK covers AdMas&Chems along their complete value chains and throughout their life cycles. It will equip SMEs and industry with digital tools (either as PINK-developed solutions or by providing ways to integrate external tools) to design and optimise AdMas&Chems for innovative applications or as safer and more sustainable alternatives. This is leading to a reduction in the environmental and socioeconomic footprint of the AdMas&Chems industrial sectors through improved: production methods, application, durability, safety, recyclability, and circularity. The PINK modelling tools allow parallel time- and resource-efficient evaluation of alternative optimisation routes, giving industry the agility and resilience to respond to external and internal influences (e.g. new market demands for AdMas&Chems, regulatory requirements, potential shortages and/or environmental impact of raw materials). While the integrated modelling tools provided by the PINK consortium mainly focus on AdMas&Chems design, data and models for process and product design, which are the other two aspects influencing functionality, safety, and sustainability, can also be integrated, if made available by industry partners or from public sources.

### 2.1 SSbD dimensions, multi-objective optimisation and tiered approach

**Fig. 2:**
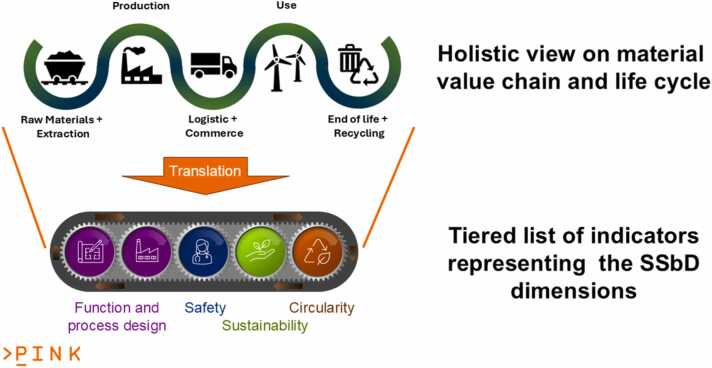
Schematic visualisation of the translation step of a linear value chain and material life cycle into indicators describing the SSbD dimensions (functionality and efficacy, resilience with respect to raw material resources and energy, physical and mechanical endurance, to human and environmental safety, recyclability, and circularity). PINK’s modelling tools, selected according to the number of AdMas&Chems candidates and data availability of the current material development stage, are then applied to predict these indicators (or a subset of these), optimise them using generative AI and provide decision support for selecting the candidates, which balance the different dimensions according to the design goals. More details on this tiered approach are given in the text. Circular and interlinked value chains add additional indicators, but this additional complexity was neglected in this figure for easier readability.

The development of SSbD AdMas&Chems requires optimisation and trade-offs across all SSbD dimensions from functionality and efficacy, resilience with respect to raw material resources and energy, physical and mechanical endurance, to human and environmental safety, recyclability, and circularity taking into account the full value chain and material life cycle (see Fig. 2) as defined in the SSbD Framework and other SSbD recommendations, e.g. by the European Chemical Industry Council (CEFIC) [47], [48] and the InteRnatIonal ecosystem for accelerating the transition to Safe-and-Sustainable-by-design materials, products and processes (IRISS) [49], [50], [51]. The SSbD Framework has meanwhile significantly progressed as described in the recently published methodological guidance [14], profiting from stakeholder-informed refinements during the two testing phases [13] and the assessment criteria and workflows for evaluating them have been customised / prioritised during the specific material design campaign. More information on PINK’s industry-focused approaches and entire-life-cycle perspective are presented in an extended perspective article [52]. We also refer to this perspective article for a discussion on how the SSbD Framework relates to previous risk assessment frameworks and industry practices as well as insights into current progress towards prospective and anticipatory life cycle assessment procedures [53], [54] and opportunities provided by machine-learning-assisted approaches to assess impact across the entire life cycle of AdMas&Chems.

With emphasis on the central role that new approach methodologies (NAMs) play in its implementation, SSbD is, within PINK, conceived as a multi-objective optimisation problem with the goal of balancing diverse requirements based on all the available data, with their associated uncertainties, at the specific stage of the AdMas&Chems development process. The term NAMs covers a range of state-of-the-art safety testing methods (see [55] for further details), both wet-lab and computational, that are not yet fully validated towards regulatory application but which offer cost-and-time-efficient alternatives to currently demanded animal testing [56]. They also show potential to be more human-relevant due to the use of human cell models or being based on human data and provide mechanistic information on the initiating event and biological responses leading to the adverse effect [56]. However, they still need to further establish their relevance and reliability to be integrated into industry settings and the regulatory framework and demonstration of their effectiveness in supporting implementation of the SSbD Framework, which is currently a voluntary standard, is a potential route to gaining broader regulatory acceptance of NAMs, both of fully computationally as well as integrated with experimental approaches, e.g. in Integrated Approaches to Testing and Assessment (IATA) [57].

**Fig. 3:**
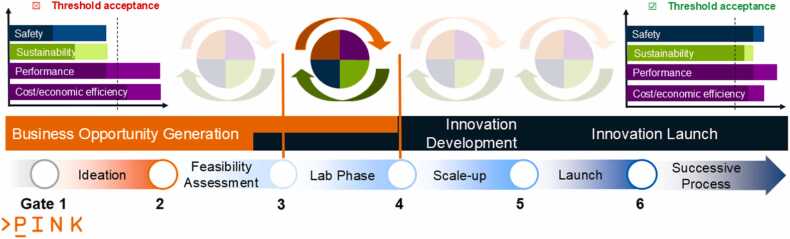
Schematic presentation of the PINK R&I Approach, in which the multi-objective optimisation, represented by the circle and the circular arrows above each stage, is performed in every development stage from ideation to launch. The case shown represents a redesign, where a performant material with a bad safety and sustainability profile (left) is modified until all categories surpass a threshold for acceptance (right) even if some categories, cost/economic efficiency in this case, get a lower score compared to the original material. Simultaneously, the uncertainty for all categories is reduced according to the requirements of the current material development stage, which is assumed here to be lab phase. The colour code of Fig. 2 is reused with purple: function and process design including economic feasibility, green: sustainability, blue: safety and brown, circularity.

**Fig. 4:**
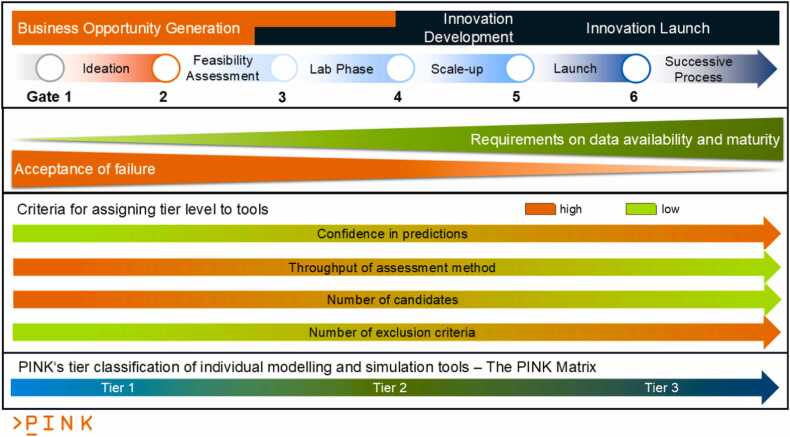
Relationship between PINK tier levels and stages of the AdMas&Chems development process and criteria used for assigning tier levels to the data and models integrated in PINK. At early stages, decisions have to be made to select promising candidates from a large number of alternatives based on limited pre-existing knowledge. High-throughput tools can be applied here, which often come with lower confidence levels. Additionally, indicators defined in the SSbD Framework as stop/exclusion criteria can often not be predicted precisely enough to justify exclusion of otherwise promising candidates. Thus, candidates showing some reason for concern can still be taken to the next stage of development since, even if these concerns manifest themselves fully later during the generation of additional data, failure is still acceptable since valuable information was produced usable for the design of new candidates. When moving to later stages (Lab Phase, Scale-up and Launch), the level of confidence needs to increase accordingly, which requires more expensive methods and validation of the computational results by generation of experimental data at least for some of the candidates.

Fig. 3 and Fig. 4 illustrate the PINK’s methodological approach: The integration of SSbD into the development process of AdMas&Chems (Fig. 3) is achieved by solving this multi-objective optimisation problem to improve and balance the four requirement categories (i.e. functionality, cost-efficiency, safety and sustainability) at each stage of the development. All SSbD dimensions including the five steps of the SSbD Framework are evaluated in each AdMas&Chems development stage, even if starting with a limited, incomplete set of indicators per SSbD category defining the current level of knowledge about the materials and rough estimates at the ideation stage (Tier 1 in Fig. 4). This is essential to provide the throughput needed for assessing all potential AdMas&Chems candidates, which might show the desired functionality profile. The low confidence in the predictions is still acceptable as long as they provide some evidence for the decision process and candidates with bad safety and/or sustainability profile can be filtered out according to the “fail early, fail cheap” principle. PINK then proposes higher-tiered methods (Tiers 2 and 3 in Fig. 4) when moving on to later AdMas&Chems development stages, providing higher confidence in the predictions. In this way, increasing amounts of information of increasing quality is produced on a constantly reducing set of better performing AdMas&Chems candidates potentially additionally complemented by experimental validation. To be able to suggest the right tools at the right time, a categorisation of PINK data and modelling resources has been performed and will be repeated for each newly integrated computational as well as experimental methods, which can provide relevant data for decision making. Note that tier categories have no hard boundaries in PINK and a tool predicting a specific indicator can be assigned to two or even three tier levels based on evaluation of throughput, data needs and level of confidence.

### 2.2 The PINK platform, interoperability and expandability

However, developing individual advanced computational models for every indicator is not sufficient to realise the vision of a digitalised SSbD. Industry and especially SMEs, as the main drivers in the development of innovative AdMas&Chems and their market introduction, need, besides easy and direct access to data and knowledge and tools for filling existing information and knowledge gaps, an infrastructure to integrate all these tools in a harmonised and interoperable way as well as user interfaces providing advanced data visualisation option, tools to explore the chemical/material space and data-driven decision support leveraging all available data to guide the design of AdMas&Chems. To achieve this, PINK creates an open innovation platform, the PINK Platform, and dedicated user interfaces, the PINK *In Silico* Hub (PINKISH). For driving the platform, PINK is providing two key innovations:

(i) An Interoperability Framework for harmonising data, models and software (which are currently still largely developed independently) across all dimensions giving FAIR and as-open-as-possible access to all information generated through data mining, modelling, simulations and AI technology, including sensitivity and uncertainty analyses; and
(ii) Innovative ways to provide decision support, which combines advanced data analysis and visualisation (i.e. the PINK Dashboard) with advanced artificial intelligence (AI) technologies (deep learning, causal and generative AI) during the complete design process (i.e. from design ideas to registration and market entrance), based on a tiered approach. This approach aims to optimise and balance both throughput and confidence in the computational model according to the AdMas&Chems development stage.

All resources, both from PINK partners and from third parties, to be implemented in the PINK Platform are (i) being evaluated for their usability and practicability in industrial settings, (ii) undergo improvement on their technical and semantic interoperability, and user experience in collaboration between the PINK partners and the original developers, and (iii) are combined to provide the input for data visualisation of the complete set of SSbD indicators in the PINK Dashboard and decision support for AdMas&Chems development via PINKISH. Integrated tools are also modified in a stepwise manner to be able to exploit the newest web and cloud technologies to generate a secure environment, according to the highest industry standards for intellectual property protection, access rights and ethics. Additionally, integration of local data, models and computer infrastructure for highly sensitive data is made possible.

The innovative potential of the platform is validated in real-world scenarios, by conducting two types of industry-driven studies: (a) large-scale Developmental Case Studies first, and (b) Industrial Demonstrators later (mainly conducted by SMEs, which will be recruited via an open call for participation). The Developmental Case Studies guide the platform's development and integrate industry expectations on functionality, practicality, user experience, and software/data security. The Industrial Demonstrators, by contrast, will integrate additional industrial end users into PINK, via the private-side association IAM-I of IAM4EU (and potentially its external advisors from ECETOC, European Polymer Dispersion and Latex Association (EPDLA) and the Circular Economy at the Chamber of Commerce and Industry of Štajerska). These new partners will be supported by the complete consortium in stress test the PINK solutions and boost SSbD implementation in SME-led research and innovation.

The data and modelling resources and the PINK Platform are meant to be generally applicable. While the PINK consortium covers all the needed expertise to develop and provide the tools, it was obvious from the proposal stage that not all AdMas&Chems classes and markets can be covered by PINK-developed tools alone. Recognizing this limitation, the list of tools and the Platform are being developed to be extendable and future-proof to ensure their utility well beyond the project’s runtime. Therefore, a considerable number of the activities are designed to guarantee that PINK does not stand alone, but is fully integrated into the evolving European ecosystem of initiatives (including EU strategies, other initiatives and projects) implementing the twin green & digital transition following the SSbD Framework. PINK partners have already established collaborations with IRISS and the Partnership for the Assessment of Risks from Chemicals (PARC) [58], [59] and is involved, via PINK consortium partner IAM-I in the creation of the IAM4EU Partnership. Together with related projects, including various Open Innovation Test Beds, DigiPass [60] and several other SSbD methodology developing projects, PINK is actively establishing bridges between the chemicals and materials worlds, specifically focusing on the needs of industry and more specifically SMEs. These collaborations are based on openness and FAIRness [43], [45], [46], [61], which lie at the heart of PINK. Following these principles is essential for the smooth operation of the developed platform and its integration into the broader AdMas&Chems data, modelling and software ecosystem [4]. PINK’s Interoperability Framework is a response to the existing fragmentation of models, software and tools and is meant to break up data silos, which exist due to the hitherto largely independent developments of the fields of (i) chemical design, (ii) materials modelling, (iii) characterisation, (iv) and safety and life-cycle assessment. Sharing the concepts implemented in the Interoperability Framework and the corresponding infrastructure tools (see below) with all relevant communities and stakeholders fosters harmonisation of the FAIRification approaches including (meta)data standards, information transfer formats, and persistent identifiers, as well as generating a common data documentation environment that builds on and maps established ontologies and data models within disciplines and across domains [62], [63].

## 3 Road to innovation

PINK’s methodology approach is based on existing material design principles to guarantee functionality and economic feasibility while meeting criteria for safety and environmental sustainability as proposed in the SSbD Framework. Due to its industry focus, PINK prioritises usability, practicality and affordability in industry settings, especially in SMEs that lack dedicated modelling capability, ensuring that data and models are suitable for commercial use, and optimising user experience design and data security according to industry standards (e.g. ISO/IEC 27001: Information Security Management Systems, ISO/IEC 27002: Code of Practice for Information Security Controls, ISO/IEC 27018: Protection of Personally Identifiable Information (PII) in Public Clouds and ISO/IEC 27032: Guidelines for Cybersecurity).

Computational models, software tools and data for molecular design, materials modelling and characterisation, safety and sustainability including LCA [64], [65], [66], [67] and Life Cycle Costing (LCC) [68] have already been developed by members of the PINK consortium and third parties. Specifically for LCA, the perspective article written by the PINK consortium and also published in this “MaterialsWeek 2024” special issue [52] provides a general understanding of LCA methodology used in PINK including an insight into prospective and anticipatory LCA. Additional tools have been developed in contiguous communities such as the materials modelling community represented by the European Materials Modelling Council and the chemical and advanced materials safety and sustainability communities as organised in SUSCHEM, the EU NanoSafety cluster, NanoFabNet [69], and others. However, the tools are scattered and sit in silos both within communities and across different communities. Therefore, one of the primary objectives, as described above, is the development of an open technical and semantic Interoperability Framework, which, on one hand, builds the basis for the developed platform, tool and data integration and decision support, and, on the other hand, is intended to provide a blueprint for a generally applicable approach for AdMas&Chems’ SSbD infrastructures that spans the relevant communities, positioning PINK as a prototype for the infrastructure needed to support the European materials data / digital ecosystem. The Interoperability Framework will provide the flexibility to ensure the sustainability of the platform itself and its future extendibility. PINK will provide modelling tools mainly for material and process design while product design aspects is only addressed through the inclusion of data provided by industry partners or through modelling related to the Developmental Case Studies. However, options for onboarding additional models and data sources for all design dimensions, (i.e., allowing third parties to provide tools as part of the Platform), will be explored during the project’s runtime, to be fully implemented by its end.

### 3.1 Comparing the PINK approach to the SSbD Framework

As briefly introduced above, the SSbD Framework proposes two high-level components, which are executed iteratively: (i) the (re-)design phase, and (ii) the safety and sustainability assessment phase, which is further divided into 5 steps. PINK will follow the guidelines and recommendations of the SSbD Framework and further increase their utility by implementing the evaluation of the SSbD indicators into a flexible computational SSbD workflow and an AI-driven decision support system (DSS) exploiting computational NAMs as well as data retrieved from *in vitro* NAMs, integrated approaches for testing and assessment (IATAs) and automated data curation from databases and literature. To acknowledge the need of industry, and specifically SMEs, PINK deviates slightly from the workflow concepts outlined in the SSbD Framework in three aspects:

1. The strict separation between the (re-)design and assessment phase has been removed: High-throughput computational approaches are fast enough to not only evaluate the final candidates but allow exploration of a multitude of design routes and (virtual) AdMas&Chems candidates. Therefore, specific indicators from the first four assessment steps (with Step 5 indicators to be added when available) will be evaluated together with functionality and economic viability using a multi-objective optimisation procedure;
2. The workflow is flexible to the input it accepts: This allows for specific indicator lists for different stages of the AdMas&Chems development process and future updates of these lists whenever new requirements from industry or regulatory guidelines and standards are introduced. Integration of additional parameters from process and product design is also possible (onboarding of third-party modelling methods). Flexibility to select and weight indicators according to the specific type of innovation and AdMas&Chems class is provided;
3. The use of cut-off criteria to stop further evaluation is only enforced in later development cycles: In the SSbD Framework, reasons for concern from one indicator (e.g. hazard category 1 A for carcinogens or even the lack of information about this endpoint) may terminate the process without considering other criteria. However, multiple candidates should be characterised in the early stages to understand the influence of chemical and structural variations on functionality and sustainability even if early evidence (or lack of data) points to safety concerns.

**Fig. 5:**
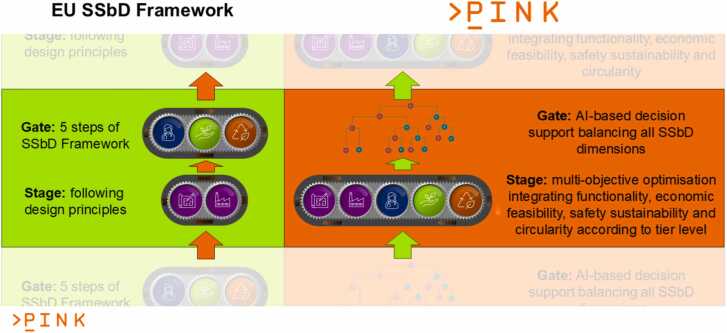
Schematic representation of the PINK approach (right) compared to the hierarchical approach described in the SSbD Framework (left). The separation between evaluation of functionality and economic feasibility in the stage phase and full assessment of safety and sustainability in the gate phase is replaced by performing a multi-objective optimisation of all SSbD categories in parallel and providing the results to the DSS. After this is completed for one stage of the AdMas&Chems development cycle, this process is repeated using additional information from more expensive computational and experimental (wet-lab) methods.

Considering these three modifications to the SSbD concept, each AdMas&Chems development stage is supported in the PINK methodological approach as depicted in Fig. 5. Each development cycle starts with data mining and data creation for functionality, safety, sustainability, and cost/economic feasibility inputs, which are provided simultaneously to the decision support workflow, which is described in detail in the next subsection. The specific indicators and methods used in the evaluation depend on (i) the number of AdMas&Chems candidates considered in a specific development stage (ranging from many at the idea generation stage to a few (or just one) at the market introduction stage) and (ii) the knowledge depth availability about the candidates (only predictions for only virtually existing candidates at the idea generation stage versus full assessment of the final candidate ready for market introduction).

In Fig. 5 only one stage-gate cycle is shown e.g. representing the Lab Phase in Fig. 3. Moving on in the AdMas&Chems development process is symbolised by the green and orange arrows. Fig. 4 already presented how different properties describing the knowledge about the AdMas&Chems candidates and requirements on the used methods advance as the development project moves from lower to higher tiers. To quickly recapitulate: the indicators to be included and the methods to evaluate them depend on the number of AdMas&Chems candidates identified at each development stage and the knowledge about them. The PINK Tiered Approach categorises all computational methods into three tiers that parallel the AdMas&Chems development stages, often also expressed as material technology readiness levels (TRLs), with respect to their data needs, throughput, and confidence levels. At early development stages (ideation/design phase of AdMas&Chems development), where less data is available, high-throughput computational approaches of low tiers are applied to avoid limiting the exploration and missing promising design routes due to time constraints (human and computer) and costs. This allows consideration of promising design routes and candidates, which can then be further investigated using more expensive but also the more accurate methods of higher tier. Suggestions for experimental data generation (*in vitro* NAMs and later *in vivo* and pilot-plant level studies) as input for the next cycle are also proposed, for example through mechanistic investigations aligned with Adverse Outcome Pathway (AOP) concept [70], [71] combined with frameworks that can guide data collection and/or generation through e.g. IATAs [72], [73]. We refer to the literature for a detail description of these approaches [71], [72], [74] and to the Innovation Report of the INSIGHT project describing the integration of sustainability aspects in Impact Outcome Pathway [75]. Finding appropriate methods for all tiers will be key to the success of this methodological approach and the SSbD Framework overall.

### 3.2 Computational approaches and the decision support system

**Fig. 6:**
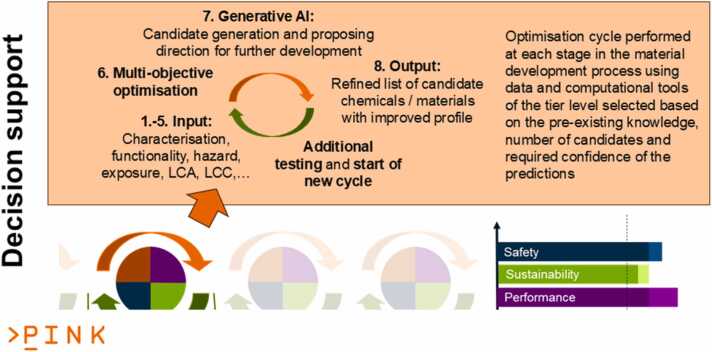
Schematic presentation of the eight steps of the decision support workflow with step 1–5 producing the input data for the multi-objective optimisation of step 6. Especially in early AdMas&Chems development stages, step 7 generates additional candidates to further explore the chemical/material space. Step 8 visualises the output to give the user all the information to select the candidates to take over to the next development stage.

In the current first phase, PINK is collecting existing approaches and complementing these with new developments for the evaluation of functionality, safety, and environmental sustainability indicators based on physics-based and data-driven/AI computational approaches, LCA, AOP-based knowledge extraction as well as combinations of these. Additionally, data sources and text-mining approaches are made available. All information produced by the predictive models or data mining methods are integrated into the PINK Platform and will be combined with causal AI and generative AI approaches for the design of new AdMas&Chems candidates to support the candidate-selection decision-making process in the second phase of the projects. Users will then be able to visualise all relevant data in the PINK AdMas&Chems Dashboard and follow the data provenance trail to access all details on how the data was generated, what assumptions were made and what the expected confidence levels associated with the results are. Additionally, users have the ability to select the indicators that are most relevant to their specific objectives and define their importance (weightings). The selected indicators will be used by the multi-objective optimisation process and generative AI technologies to design and select optimal candidates [76], [77]. At the proposal stage, the final workflow of the DSS was envisioned to include eight steps, which are currently being refined and evaluated for their applicability to the Developmental Case Studies. The eight steps are (see also Fig. 6):

1. Define the criteria that are used to optimise and evaluate different AdMas&Chems candidates based on the four requirement categories (functionality, cost-efficiency, safety, and sustainability) and possibly assign weightings for the importance of each indicator to guide the multi-objective optimisation.
2. Search for existing information on similar AdMas&Chems to the substance of interest, using AI techniques such as graph-based methods or fingerprint similarity analysis.
3. Depending on the volume of available data, apply read-across methods or machine learning (i.e., Quantitative Structure Activity / Property Relationship (QSAR/QSPR) models) to predict functionalities and SSbD properties of similar AdMas&Chems and select the most promising ones, according to their weighted performance with respect to the optimisation criteria.
4. Use mechanistic multi-scale simulations [78] to predict functionalities and interactions between selected AdMas&Chems and their environment that cannot be predicted using data-driven models.
5. Conduct (prospective and anticipatory) LCA of selected AdMas&Chems to evaluate their environmental impact across their entire lifecycle, including production optimisation, use phase, and disposal stage.
6. Perform real-time evaluation and comparisons of AdMas&Chems candidates based on their predicted functionalities, LCA-based evaluations and SSbD characterisation utilising the data visualisation tools available in the PINK Dashboard to guide the identification of the most important indicators to be used in the selection of candidates to proceed with.
7. Leverage the power of generative and causal AI to explore new and innovative AdMas&Chems design options, beyond the limitations of existing data. By incorporating property constraints, the decision support workflow will generate novel structural and potentially biological (based on omics experiments) fingerprints, evaluate their chemical feasibility, and propose new promising AdMas&Chems designs for the specific application. Generative AI (e.g. variational autoencoders [79] or generative adversarial networks [80]) will provide an efficient and effective way to explore the vast chemical space and discover the most suitable AdMas&Chems for further development. causal AI techniques [81], [82] (e.g. causal Bayesian networks [83] and double/debiased machine learning [84]), will identify areas of improvement to assist in modifying the structure and designing new AdMas&Chems of improved specifications.
8. Present a final shortlist of selected AdMas&Chems to the user, along with the optimisation criteria and the respective evaluation metrics, in easy-to-interpret formats like tables or spider graphs. “Final” is meant here only regarding the current execution of the multi-objective optimisation in one AdMas&Chem development stage. A multi-decision support algorithm ranks the AdMas&Chems included in the final list, according to the optimisation criteria. Furthermore, at this stage, the DSS will provide recommendations and tools for additional computational and experimental testing to be performed in the next AdMas&Chem development stage to increase confidence in the predictions and perform further higher-tier analysis on the most promising AdMas&Chems candidates.

The output of the decision support workflow is expected to be presented in a form similar to the scoring system recommended in the SSbD Framework [11], [12], [14] and applied in the case studies of the testing phases [13]. As already mentioned above, also other easy-to-understand visualisations like spider graphs and the Dashboard, giving access to all information for a specific chemical/material including structural and physicochemical characterisation with links to descriptions of the used methods and the raw data, are being provided. However, it is also important for decision making that the user can explore the relationships between the data and how these influence the SSbD classification of a candidate compared to similar chemicals/materials. To provide easy access to such relationship information and offer functionality for further analysis, PINK is collaborating with the INSIGHT project [85] to define the requirements and strategies to realise an Integrated Knowledge Graph for Chemical Impact Assessment. This represents the first application of knowledge graphs in PINK, while the second is the PINK Knowledge Base (PINK KB) indexing data resources and tools as described in Section 3.3. A Knowledge Graph (KG) is a structured data representation, in which entities are depicted as nodes and the relationships between them as edges. This interconnected system facilitates the integration, retrieval, and analysis of diverse and heterogeneous data from multiple sources. Due to their ability to systematically organise complex interrelations and enhance data reusability, KGs offer significant potential for advancing holistic approaches in chemical safety assessment [86].

By structuring chemical entities as nodes and their interrelationships as edges, the KG for Chemical Impact assessment includes heterogeneous data pertaining to the chemical structures, properties, and endpoints necessary for assessing human and environmental health impacts, as well as social, economic, and environmental sustainability of AdMas&Chems. Besides data generate during the decision support workflow, natural language processing techniques are employed to extract relevant connections between chemicals and endpoints from scientific literature. Multi-dimensional read-across analyses through link prediction is enabling the extrapolation of properties for under-researched AdMas&Chems based on established knowledge from well-characterised substances. More information on the use of KGs in the development of the Impact Outcome Pathway concept is available from Serra at al. [75].

### 3.3 Interoperability framework and infrastructure components

Technically, PINK is based on a distributed infrastructure, where individual data and modelling resources are implemented as independent (web) services. However, it is also exploring ways to improve user experience by providing a unified interface with a common look and feel for the offered SSbD services available via the user interface PINKISH and whenever possible to the underlying data and modelling services. Web-based user interfaces are being set up to provide user and resource management and access to the SSbD decision support workflow, data visualisation and AI-enabled suggestions of potential development routes. Additionally, PINKISH will offer semantic search functionalities to enable identification of relevant data resources and modelling tools according to the AdMas&Chems class of interest and tier level. Finally, PINKISH will provide access to the a dashboard summarising all data generated in the current and all previous cycles of running the SSbD workflow and the results of the SSbD assessment and conclusions/decisions based on the holistic assessment generated by the modelling tools and complemented by input from the human (material design, safety and sustainability) experts.

**Fig. 7:**
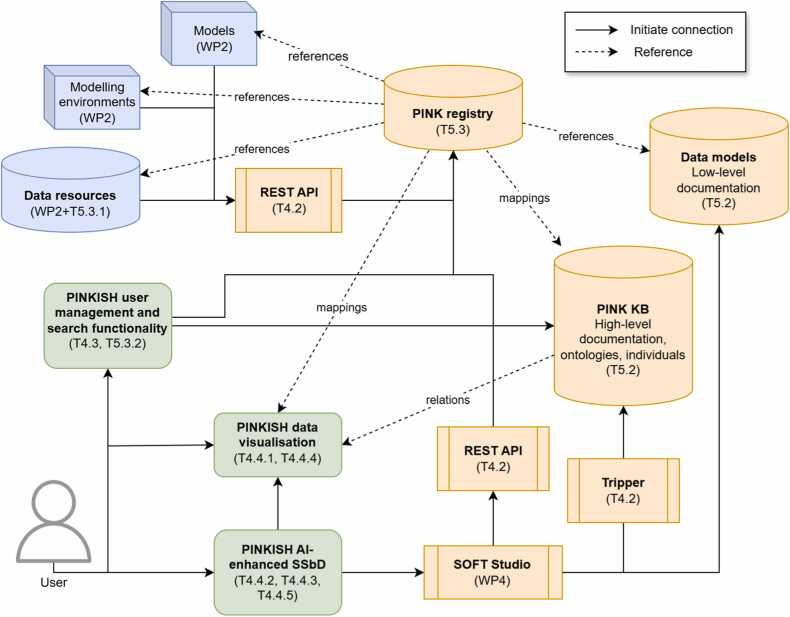
Illustration of the architecture of the distributed PINK infrastructure including the independent data and modelling services (blue), the PINKISH components for data visualisation and SSbD decision support (green) and the semantic and technical interoperability framework (orange).

A schematic representation of the modelling and interoperability software stack that comprises the PINK Platform including PINKISH is presented in Fig. 7. To integrate the different data, models and software, as well as the services built on top of these (Dashboard, DSS and generative AI) into one Open Innovation Platform, a major part of PINK is devoted to achieving interoperability. This is based on data and application programming interface (API) documentation and annotation performed on multiple levels and implemented in a stepwise manner to profit from semantic integration as soon as possible. Data resources, models and tools are documented in the PINK KB, that also serves as a data catalogue, enabling findability and accessibility also called discoverability. This cataloguing is based on the Data Catalog Vocabulary (DCAT) [87] to ensure compatibility with an already widely adopted standard for specifying e.g. where a data and modelling resource can be found, and which restrictions apply to it.

**Fig. 8:**
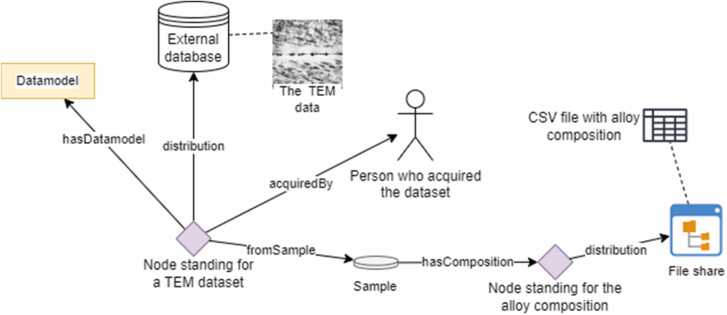
Example of how a TEM dataset might be represented as a node in a knowledge base and it relates to other nodes/resources. Ontologies are used behind the scenes to describe what the resources really are.

Furthermore, reusability is enhanced by the inclusion of context information and creating linked data, such as how datasets are related. Semantic documentation with ontologies further enhances the description by including domain knowledge, thus providing understanding and semantic interoperability. Numerical interoperability and information about the meaning and structure of the (meta)data is provided within a common interoperability system (DLite [88]), ensuring computer (machine) actionability and enabling (semi-)automatic workflow generation. Linking data models and their properties to the ontology ensures full semantic documentation of the data and thus provides meaning, which is necessary for cross-domain interoperability [89]. The finally resulting Interoperability Framework consists of a system for documenting data resources and creating formalised, semantically annotated datasets and models enabling full interoperability across domains via mapping the DLite datamodels to ontological concepts. The underpinning ontologies provide the common vocabulary with context and require rigorous development. Fig. 8 shows an example of how a TEM dataset is documented in a knowledge graph. Existing chemical, material, biological and medical domain ontologies [62], [90], [91] are utilised and will only be further developed and complemented by new domain ontologies or ontology modules where necessary. Another focus of PINK is the harmonisation and alignment of different existing ontologies to allow communication across various domains, through establishment of FAIR mappings and crosswalks [92].

The resulting semantically annotated data resources and APIs are able to automatically provide the input for (i) the PINK KB, which indexes all available services and data resources, both publicly available as well as confidential when deploying PINKISH as an in-house software, (ii) workflows combining different services to evaluate complex criteria (e.g. using physics-based simulations to provide input for LCA or implementing IATAs), (iii) KG construction to provide relationships in the PINK KB as well as the Integrated KG for Chemical Impact Assessment, and (iv) the components of PINKISH, the DSS workflow (for the evaluation of candidates), the generative AI (for innovative AdMas&Chems design) and the Dashboard (for data visualisation and references to method description and raw data).

### 3.4 Developmental Case Studies and Industrial Demonstrators

Data and modelling tools and solutions are developed to directly support SMEs and industry via the Platform, the data and modelling services and PINKISH. To ensure that the platform addresses all requirements and conforms to industry standards concerning functionality, user experience, data and software security, the developments are guided first by two Developmental Case Studies and will be followed by a set of Industrial Demonstrators. The Developmental Case Studies were selected based on the market relevance of AdMas&Chems and data availability to cover multiple cycles of the development process. Since PINK intends to provide the tools to perform SSbD rather than engaging in AdMas&Chems development itself, the case studies are based on previous activities performed by the industry partner BASF and are focussed on evaluating the integration of safety and sustainability aspects early into the AdMas&Chems design process. This has the advantage that a rich data and information basis (with varying complexity and confidence levels) on potential alternatives and documentation of the decision process on how these alternatives were selected is available as an input to model the design process. Available data that feeds into the Developmental Case Studies include read-across from similar substances for early design stages up to *in vitro* data from toxicity testing and production parameters from lab settings and even pilot plants for the later almost final design stages.

With respect to the first three steps of the SSbD Framework (hazard assessment of chemical/material; exposure assessment during the chemical/material production and processing phase, and human health and environmental aspects in the final application phase), the available data used in the Developmental Case Studies is helping to evaluate the pros, cons and limits of the range of models and tools that are integrated into the Platform. Due to their broad application areas, the two selected Developmental Case Studies bridge the chemicals and advanced materials sectors. Furthermore, datasets on risk assessment within the production and processing phase, consumer use and environment (including exposure, release, and fate data) are sourced, and data gaps identified. Additionally, LCA and social assessment (i.e. steps 4 and 5 of the SSbD Framework) are being performed for both Developmental Case Study substance classes (i.e., styrene/acrylate copolymers and organic UVB). Besides these two PINK Developmental Case Studies, we explore the possibility of running additional studies in close collaboration with other projects and work together with the EU JRC to provide the results to the EC as a continuation of the testing phase of the SSbD Framework supporting its refinement and broader adoption.

The Platform is constantly being improved and optimised based on the requirements imposed by the Developmental Case Studies in an iterative cycle. Feedback is used to define which indicators representing functionality, safety and sustainability assessment can be introduced at which stage of the AdMas&Chems development process and what level of confidence is needed at each stage to guide the selection of candidates to forward to the next cycle. Thus, the Developmental Case Studies (i) refine the categorisation of the methods into tier levels, (ii) select which criteria the DSS can rely on and (iii) how data gaps can be filled by new methods and models at each specific stage. By performing the Developmental Case Studies in collaboration with model innovators, platform developers and industry partners, they also evaluate the industry readiness of the system and support its improvement. This collaboration will ensure a balance between (i) the flexibility to be customisable to the problem at hand versus the ease-of-use and (ii) the availability of data and tools versus data security and IP protection needs.

Access to the PINK Platform and PINKISH, including the models, the DSS and accompanying consultancy and training services, will be offered in the last phase of the project as part of the Industrial Demonstrator programme. In this way, the benefits of the platform and the need for additional support will be further evaluated, the commercial sustainability of the services will be assessed, and the final business plan will be defined based on real-world implementation cases. The Industrial Demonstrators will, thus, have an important influence on the ways the Platform will be exploited. To be able to address the real-world needs of industry at the time of execution of the Industrial Demonstrators, the Demonstrators were not pre-specified at the proposal stage but are currently being actively recruited. Discussion with different industry associations already demonstrated high interest in the new methodological approach and resulted in Energy Materials Industry Research Initiative (EMIRI), which has now been transformed into IAM-I, joining the consortium and the European Polymer Dispersion and Latex Association (EPDLA) participating in PINK’s External Advisory Board. Direct dissemination of all outputs to members of IAM-I and EPDLA is used for discussing and defining potential Demonstrators. The Industrial Demonstrators are expected to cover AdMas&Chems for (a) clean and sustainable energy & mobility (e.g. batteries, solar and wind energy, hydrogen production and storage), (b) low-carbon industries, and (c) water-based polymer dispersions and lattices. This does not exclude additional external users interested in becoming early adopters of the PINK Platform from receiving access and support, further increasing the industry sectors covered.

## 4 Discussion

Since PINK started in January 2024 (just over one year before writing this innovation report), the focus has been on the identification of important indicators for all dimensions required for the holistic SSbD development of AdMas&Chems via a multi-objective optimisation. For all these indicators, existing models developed by PINK partners or publicly available are collected and analysed with respect to their applicability domain, throughput and confidence levels and assigned to one or multiple tier levels. In contrast, development of the PINK Platform and its user interface PINKISH, integration of data, models and tools and the development and implementation of the Interoperability Framework and corresponding infrastructure components are currently still in the design, specification and prototype stage. Nevertheless, success in achieving the impacts outlined above can already be envisaged at this early stage of the project.

Multiple market sectors and their value-creating & -adding stakeholder communities are being supported and connected through a network of data resources and modelling tools/software (data and modelling services and PINKISH modules), harmonised and made interoperable based on the advanced concepts of the Interoperability Framework. Since usability and practicability of the provided solutions are paramount, this modular approach balances ease of use with flexibility and customisability to specific AdMas&Chems classes and applications. New data visualisation to facilitate data and knowledge exploration, improve reusability and enhance transparency and trust (in the data and tools) are provided and fully integrated in the form of the AdMas&Chems Dashboard. The decision process is further supported by the AI-enabled DSS, which is (a) guided by user input defining optimisation goals and weighting the different SSbD criteria and, (b) fully transparent for internal but also external evaluation by documentation of the process and provision of full provenance trails for the underlying data. Onboarding of new and improved approaches will increase the applicability and enable integration of SSbD indicators resulting from changed recommendations and regulations. Together with further integration of process- and product-design aspects, this will ensure sustainability of the Platform into the future.

The computational approaches of the data and modelling services and DSS provided by PINKISH significantly reduce the costs of applying SSbD to the AdMas&Chems development process (especially at its onset) and enable SMEs to realise innovative design ideas for all innovation markets identified by AMI2030 (i.e. health and medical, construction, new energies, transport, home & personal care, packaging, agriculture, textiles and electronics appliances), and beyond. The user will be able to combine commercial and freely available components that provide the most cost-effective access to solutions including (a) modelling software and predictive computational models, (b) (modular elements of) the PINK Platform, and (c) FAIRified & harmonised ontologies. This will support industry (SMEs and large) in replacing expensive *in vivo* and *in vitro* regulatory safety evaluations with digital solutions (i.e. cost-effective computational approaches including read across and AI-driven data-gap-filling solutions such as text mining, often based on open-source software and public and FAIR data resources), thus providing a flexible tool for all stages of the AdMas&Chems development process including the initial, pre-budget planning. Training materials and skills development offerings during and beyond the project lifetime provide easy starting points to help users get familiar with the PINK Platform and PINKISH, the parameters and computational approaches for the four SSbD categories, and their underlying synergetic effects. This will ultimately allow industrial users to acquire such expertise in-house, potentially without the need to establish a separate modelling division.

Beyond development of its main platform and modelling tools, central activities of PINK include (a) the harmonisation and documentation of data, models, the APIs provided to access them and the used terminology, and (b) standardisation of the FAIRification approaches as part of the Interoperability Framework. This ensures the consolidation of hitherto siloed data (i.e. the currently separate use-categories of (a) materials modelling, (b) safety, and (c) sustainability, including socio-economic modelling. Availability of all these resources (data and models) is enabling industries in all sectors to (i) evaluate the performance of AdMas&Chems according to the four requirement categories (as currently defined by the SSbD Framework and extendable to new criteria in the future) and (ii) improve the AdMas&Chems’ performance (with regard to some or all of the SSbD categories), by identifying promising candidates and suggesting alternative (virtual) AdMas&Chems for further evaluation in an iterative cycle.

Industry and SMEs, including our partners BASF and IAM-I (via its members) and external advisory board members (EPDLA and ECETOC), have started to implement SSbD approaches and industry associations like CEFIC have proposed first SSbD recommendations even before the EU Framework had been published. PINK builds on the knowledge and expertise of these industrial partners and advance their implementation of SSbD for AdMas&Chems processes and products in multiple value chains. The transferability of the innovative approach to a much wider range of AdMas&Chems-based processes and products is being established through (i) the Industrial Demonstrators, and via the short-term dissemination, communication and exploitation measures conducted during the project’s run time and (ii) the medium- and long-term technical sustainability planning combining open access to underlying infrastructures with cost-effective purchase options of both licenses and consultancy services.

PINK’s onboarding model is central to the broadening of the SSbD exemplary results to a much broader range of industries, by essentially fostering the creation of a community of innovative software and model integrators. The publicly available Interoperability Framework created by PINK provides a cornerstone to the further development of this community and forms a prototype for infrastructure components of the European materials data ecosystem. Through the onboarding process, more aspects of process and product design can further be integrated by collaboration with third parties during and increasingly after the end of the project. Over time, the foundations laid in PINK will provide a complete set of functionalities to develop new AdMas&Chems, production processes and products for optimised applications to support multiple EU action plans.

## 5 Conclusions

Addressing global challenges and reaching Europe’s goals of reducing dependence on critical raw materials, lowering energy consumption and greenhouse gas emissions, and achieving zero pollution and a toxic-free environment, requires new materials and chemicals that combine advanced functionality with safe and sustainable. PINK set out to facilitate the development of such SSbD AdMas&Chems by providing state-of-the-art digital solutions to industry and SMEs applicable to all innovation markets.

Starting with the SSbD Framework proposed by the EU JRC and endorsed by the EC as well as SSbD recommendations from industry associations, PINK transforms the SSbD Framework from a stepwise process performed iteratively into a multi-objective optimisation problem by evaluating functionality, safety, sustainability, and cost-efficiency simultaneously. Data and modelling tools and software covering the SSbD dimensions functional design, safety assessment and LCA/LCC are being developed by the PINK consortium, integrated into the PINK Platform and complemented by third-party model and tools either integrated by a PINK partner (interfacing) or by the tool owner (on-boarding). The foundation for the used LCA methodology, especially prospective and anticipatory LCA, and an overview of existing approaches for implementing SSbD-like procedures in several different industrial sectors is presented in more detail in the perspective article also published in this “MaterialsWeek2024” special issue [52].

The specifically designed technical and semantic Interoperability Framework facilitates integration, onboarding and FAIRification of all types of (currently disparate) data and modelling resources. Within PINK, the machine actionability resulting from this Interoperability Framework drives advanced data visualisation, knowledge representation and generation with the help of KGs, computational workflow generation, AdMas&Chems candidate generation using generative AI and finally an AI-driven DSS accessible via the user-facing interface, i.e. *PINKISH*. However, the main purpose of the Interoperability Framework is to achieve cross-domain and cross-platform interoperability so that PINK-compatible data and modelling resources can easily be transferred from one open innovation platform to another and can be combined in customised, user-defined ways (mix and match).

To ensure that these innovative developments can guide industry researchers through the complete AdMas&Chems development process from early idea creation to market introduction and provides results of a quality acceptable for decision making and regulatory application, two industry-driven Developmental Case Studies guide the iterative platform development process. Additionally, the Industrial Demonstrator programme will be introduced in the final project phase, which allows industry partners especially SMEs to become part of the consortium and gain direct access to the full team of experts providing support to tackle real-world SSbD challenges of these new partners. Success stories of these Case Studies and Demonstrators will be presented in additional mid-term and final innovation reports in this journal. These updates will also highlight how the collaborations initiated by PINK and more specifically the data, modelling and digital infrastructure solutions and the Interoperability Framework contributed to the generation of a European data/digital ecosystem.

## CRediT authorship contribution statement

**Nymark Penny:** Writing – review & editing, Writing – original draft, Supervision, Methodology, Funding acquisition, Conceptualization. **Marvuglia Antonino:** Writing – review & editing, Writing – original draft, Supervision, Methodology, Funding acquisition, Conceptualization. **Afantitis Antreas:** Writing – original draft, Project administration, Methodology, Funding acquisition, Conceptualization. **Sarimveis Haralambos:** Writing – review & editing, Writing – original draft, Supervision, Methodology, Funding acquisition, Conceptualization. **Furxhi Irini:** Writing – review & editing, Validation, Methodology. **Greco Dario:** Writing – original draft, Supervision, Methodology, Funding acquisition, Conceptualization. **Serra Angela:** Writing – review & editing, Validation, Methodology, Conceptualization. **Serchi Tommaso:** Writing – original draft, Methodology, Funding acquisition, Conceptualization. **Larrea-Gallegos Gustavo M.:** Writing – review & editing, Validation, Methodology. **Costa Anna L.:** Writing – original draft, Methodology, Funding acquisition, Conceptualization. **Mercuri Francesco:** Writing – original draft, Supervision, Methodology, Funding acquisition, Conceptualization. **Hagelien Thomas F.:** Writing – original draft, Methodology, Conceptualization. **Lynch Iseult:** Writing – review & editing, Writing – original draft, Supervision, Methodology, Funding acquisition, Conceptualization. **Dokler Joh:** Writing – original draft, Supervision, Project administration, Funding acquisition, Conceptualization. **Gavillet Jérôme:** Writing – review & editing, Validation, Methodology. **Exner Thomas Eckart:** Writing – review & editing, Writing – original draft, Supervision, Resources, Project administration, Methodology, Conceptualization. **Schillinger Eva-Kathrin:** Writing – review & editing, Validation, Supervision, Methodology. **Watzek Nico:** Writing – review & editing, Writing – original draft, Validation, Methodology, Conceptualization. **Friis Jesper:** Writing – review & editing, Writing – original draft, Methodology, Funding acquisition, Conceptualization. **Hischier Roland:** Writing – original draft, Supervision, Methodology, Funding acquisition, Conceptualization. **Bleken Francesca L.:** Writing – review & editing, Writing – original draft, Supervision, Funding acquisition, Conceptualization. **Gkoutos Georgios V.:** Writing – original draft, Supervision, Methodology, Funding acquisition, Conceptualization. **Seitz Christian:** Writing – review & editing, Visualization, Validation, Methodology. **Haywood Alexe L.:** Writing – review & editing, Validation, Methodology. **Karwath Andreas:** Writing – original draft, Methodology, Funding acquisition, Conceptualization. **Friedrichs Steffi:** Writing – review & editing, Writing – original draft, Visualization, Project administration, Methodology, Funding acquisition, Conceptualization. **Wiench Karin:** Writing – original draft, Supervision, Methodology, Funding acquisition, Conceptualization. **Himly Martin:** Writing – review & editing, Writing – original draft, Supervision, Methodology, Funding acquisition, Conceptualization.

## Declaration of Competing Interest

The authors declare to have no conflicting interests with the content of the study, have read and revised the manuscript carefully, agreed to its submission, and accepted their responsibility for the content.
